# The science of love is not quite there…

**DOI:** 10.1128/msystems.01189-25

**Published:** 2025-10-22

**Authors:** Adam Bode

**Affiliations:** 1 School of Archaeology and Anthropology, The Australian National University 444625 , Canberra, Australian Capital Territory, Australia; CNRS Delegation Alpes, Lyon, Rhône-Alpes, France

## LETTER

As a romantic love researcher, I was excited to see Robinson et al.’s (1) hypothetical mini-review outlining the potential microbial-endocrine interplay in love-associated emotions. I have myself considered this potential link and am pleased to see that experts have given it serious thought and presented initial ideas in the peer-reviewed literature. However, the science of romantic love is not sufficiently advanced at this juncture to be able to make some of the claims in the article.

One shortcoming of the article relates to the article’s reference to Fisher’s (2) model of reproductive emotion systems as a heuristic to consider the microbial-endocrine interplay in love. This model was postulated before a single biological study of romantic love had been undertaken, misrepresents the mechanisms and functions of the neural system involved in human reproduction (3, 4), and contradicts contemporary understandings of emotions. Sexual desire, attraction, and attachment are not emotions; they are motivational states involving emotions (see, e.g., reference 5).

The article’s assertion that “we have a good understanding of the hormone systems that play important roles in forming emotions relating to love” (see reference 1, p. 3) is inconsistent with the state of knowledge surrounding the mechanisms of romantic love. The only neurotransmitters or neurohormones that we can confidently say play a role in romantic love are dopamine, oxytocin, and opioids (see reference 3). There is evidence from one study that nerve growth factor may play a role (6), but we only have theoretical and inconsistent empirical reasons to believe that some other factors (e.g., testosterone and cortisol) may play a role (see reference 4 for consideration of inconsistent evidence). There is theoretical and indirect evidence of vasopressin’s involvement in romantic love (7), and theoretical, but only contradictory empirical evidence (8) that norepinephrine plays a role. A list of biological studies of romantic love can be found in a review (9), and the findings have been summarized elsewhere (4).

The effect of misunderstanding the evidence supporting involvement of different neurotransmitters and neurohormones in romantic love is poignantly illustrated by the claim that “altered serotonin and its link to obsessive traits may contribute to the overpowering infatuation experienced in the early stages of love” (see reference 1, p. 6). This claim is based on a common misinterpretation of an initial endocrinological study (10), does not consider a second endocrinological study (11), and does not take into account a recent psychological study on the effects of selective serotonin reuptake inhibitors in people experiencing romantic love (12).

While I value the contribution the article has made to postulating a plausible theory about microbial-endocrine interplay in love, I feel that, even at this early stage in hypothesis formulation, it is important that theory incorporate the latest knowledge about the biological mechanisms of romantic love.

## EDITOR'S NOTE

**Ed. Note:** The authors of the original article delined to respond.
